# Influence of sulfur fumigation on glycoside profile in Platycodonis Radix (*Jiegeng*)

**DOI:** 10.1186/s13020-016-0101-1

**Published:** 2016-07-06

**Authors:** Xiao-Qing Ma, Su-Mei Li, Chi Leung Chan, Tao Su, Wei-Dong Li, Hui Cao, Wang-Fun Fong, Zhi-Ling Yu

**Affiliations:** School of Chinese Medicine, Hong Kong Baptist University, Kowloon Tong, Hong Kong China; National Engineering Research Center for Modernization of Traditional Chinese Medicine, Zhuhai, Guangdong China

**Keywords:** Sulfur fumigation, *Jiegeng*, Glycosides, Processing

## Abstract

**Background:**

Over recent decades, sulfur fumigation is becoming abused in processing some freshly harvested herbs used as both medicine and food, although it has been questioned whether sulfur fumigation will change the efficacy and safety of the herbs. One of the herbs commonly processed by sulfur fumigation is Platycodonis Radix (*Jiegeng* in Chinese). Glycosides are the main bioactive components of *Jiegeng*. Up to the present, no study has been carried out to evaluate the impact of sulfur fumigation on glycoside profile of *Jiegeng*.

**Methods:**

A rapid and versatile ultra-high performance liquid chromatography coupled with ultra-high resolution quadrupole time-of-flight mass spectrometry (UHPLC UHD Q-TOF MS/MS) method was developed for comprehensive analysis of the glycoside profiles of sulfur-fumigated and air-dried *Jiegeng* samples.

**Results:**

Twenty-three glycosides were detected in air-dried and sulfur-fumigated *Jiegeng* samples. After sulfur fumigation, the peak heights of eight glycosides, namely platycogenin A, platycodin D, platycodin D_2_, platycodin D_3_, polygalacin D, polygalacin D_2_, deapio-platycodin D and 3″-*O*-acetylplatycodin D_2_, remarkably decreased; while peak heights of five glycosides, namely syringin, lobetyolin, platycoside E, deapio-platycodin D_2_ and deapio-platycoside E, slightly increased; in addition, peaks of ten glycosides, platycodin A, platycodin C, platycodin V, platycoside C, 16-oxoplatycodin D, 2″-*O*-acetylpolygalacin D, 2″-*O*-acetylpolygalacin D_2_, 3″-*O*-acetylpolygalacin D, 3″-*O*-acetylpolygalacin D_2_, and platycogenic acid B, disappeared.

**Conclusion:**

Sulfur fumigation caused significant changes of glycoside components of *Jiegeng*. Further investigations are warranted to explore how these chemical changes occurred and whether these changes would affect the efficacy and safety of *Jiegeng*.

**Electronic supplementary material:**

The online version of this article (doi:10.1186/s13020-016-0101-1) contains supplementary material, which is available to authorized users.

## Background

Herbs used as medicines and/or foods need to be processed after harvest. One of the processing methods is sulfur fumigation, which was first applied in preparing a processed form of Dioscoreae Rhizoma (*Guangshanyao* in Chinese), in Henan province, China, in 1900. The sulfur dioxide (SO_2_) generated during sulfur fumigation can bleach the herbs, and kill contaminated/parasitical microbes and insects on the herbs thus prolonging shelf life of the herbs. However, sulfur-fumigation would inevitably change the chemical components in the herbs. Recently, the disadvantages of sulfur fumigation have drawn much attention in Chinese medicine and food sectors. Since 1995, the number of sulfur-fumigated herbs has been gradually reduced in individual editions of Chinese Pharmacopeia (CP), and the sulfur-fumigation method for processing Chinese materia medicas (CMMs) was abolished in the 2005 edition of CP. Due to the benefit of sulfur fumigation on prolonging shelf life of CMMs, some CMMs were still processed by sulfur fumigation after CP2005 was issued. In CP2010, the limits of SO_2_ residue on sulfur fumigated herbs were settled. Nevertheless, some reports have shown that sulfur fumigation could cause dramatic changes in chemical profiles of various herbs [1–8].

Platycodonis Radix (the dried root of *Platycodon grandiflorum*, *Jiegeng* in Chinese), a Chinese medicinal herb traditionally used for dissipating phlegm, is commonly processed by sulfur fumigation. To the best of our knowledge, no study has been conducted to evaluate the influence of sulfur fumigation on the chemical constituents of *Jiegeng*. Glycosides are the main bioactive constituents of *Jiegeng*, responsible for a diversity of bioactivities such as anti-inflammation, anti-allergy, antitumor, augmentation of immune responses, anti-obesity, and anti-hyperlipidemia [9–13]. In this study, we compared the compositions of glycosides in sulfur-fumigated and air-dried *Jiegeng* samples using a newly developed ultra-high performance liquid chromatography coupled with ultra-high resolution quadrupole time-of-flight mass spectrometry (UHPLC UHD Q-TOF MS/MS) method. The UHPLC with sub-2-μ liquid chromatography provided strategies to improve resolution while maintained or even shortened the overall run time. Q-TOF MS enabled automated exact mass measurement of precursor and production spectra for structural elucidation with high confidence. The UHPLC UHD Q-TOF MS/MS offered superior chromatographic resolution with exact mass measurements for both MS and MS/MS modes.

## Methods

### Chemicals

Acetonitrile is of LC/MS grade (Fisher Scientific, Pittsburgh, PA, USA) and formic acid is of HPLC grade (Sigma-Aldrich, St. Louis, MO, USA). Ultra-pure water was prepared using a Milli-Q Plus water purification system (Millipore, Billerica, MA, USA). All other reagents used for extraction are of analytical grade.

### Reference compounds

The reference compounds syringin, lobetyolin, platycodin D, platycodin D_2_, deapio-platycodin D were from Sichuan Wei Keqi Biological Technology Co., Ltd. (Sichuan, China). Sulfur, complied with the Chinese Pharmacopoeia (2010 edition), was obtained from the Chinese Medicine Clinic of Hong Kong Baptist University.

Stock standard solutions of syringin, lobetyolin, platycodin D, platycodin D_2_, deapio–platycodin D (each about 1 mg/mL) were separately prepared in methanol and were stored at 4 °C before use. A mixed reference standard solution was prepared by mixing and diluting the five stock standard solutions with methanol to get a concentration of 0.1 mg/mL for each standard. The solution was filtered through a 0.45 μm PTFE filter prior to UHPLC UHD Q-TOF MS/MS analysis.

### Herbal samples

Fresh *Jiegeng* samples were collected from Inner Mongolia Autonomous Region, China. The identities of *Jiegeng* samples were authenticated to be the root of *Platycodon grandiflorum* by morphological methods according to the monograph in CP2010 by Dr. Zhi-Ling Yu. The voucher specimens were deposited in the Technology Development Division, School of Chinese Medicine, Hong Kong Baptist University.

Sulfur-fumigated *Jiegeng* samples were prepared separately from two batches of fresh *Jiegeng* samples according to the following procedures: 10 g of sulfur powder was heated until it was burned. The burning sulfur and 100 g of fresh *Jiegeng* slices (2–4 mm in thickness) were put into the lower and upper layers of a desiccator, respectively. The desiccator was then kept closed for 12 h. The sulfur fumigation was repeated twice with each 10 g of sulfur powder. After fumigation, the sulfur-fumigated *Jiegeng* slices and its corresponding fresh *Jiegeng* slices were air dried in a fume cupboard for 6 days. The SO_2_ residues in sulfur fumigated *Jiegeng* samples were determined to be around 0.43 g/kg following the method described in CP2010.

All samples were first homogenized, pulverized in a mill, and passed through a 20-mesh sieve before analysis. The powdered sample (4.0 g) was accurately weighed in a 150 mL conical flask and extracted once with 40 mL of water by sonication (240 W) for 30 min. The extract was filtered and the supernatant was transferred to a 200 mL round-bottomed flask. The extraction procedure was repeated twice. All the extracts were combined and evaporated to dryness at reduced pressure in a rotary evaporator. The residue was dissolved in 16 mL of methanol. Diethyl ether (35 mL) was added to the methanolic solution and the precipitate was collected. The collected precipitate was extracted with methanol for three times (16, 8, 3 mL). The combined methanol extracts were evaporated to about 10 mL. The methanolic concentrate was washed twice with 35 mL of diethyl ether each time. The methanolic fraction was then transferred to a volumetric flask (10 mL) and was made up to the mark with methanol. All samples were filtered through a 0.45 μm PTFE membrane filter prior to UHPLC UHD Q-TOF MS/MS analysis.

### Liquid chromatography

The UHPLC conditions for LC–MS analysis were as follows: chromatography was performed on an Acquity UPLC HSS T3 C_18_ column, 2.1 × 100 mm i.d., 1.8 μm (Waters Corp., Milford, MA, USA); and the column temperature was maintained at 40 °C. A gradient elution of solvent A (Milli-Q water with 0.1 % formic acid) and solvent B (acetonitrile with 0.1 % formic acid) was applied as follows: 0–5 min, 10–20 % B; 5–10 min, 20–23 % B; 10–15 min, 23 % B; 15–18 min, 23–27 % B; 18–26 min, 27–32 % B; 26–26.1 min, 32–100 % B; 26.1–30 min, 100 % B. An equilibration period of 4.0 min was used between individual runs. The flow rate was 0.35 mL/min, and the injection volume was 0.5 μL.

### Mass spectrometry

An Agilent 6450 UHD Accurate-Mass Q-TOF mass spectrometer (Agilent Technologies, Santa Clara, CA, USA) was connected to the Agilent 1290 Infinity UHPLC system via electrospray ionization (ESI) ion source with Jet-Stream technology for the LC/MS/MS analysis of *Jiegeng* samples. The ESI–MS spectra were acquired in both positive and negative modes. Ultra-pure nitrogen (N_2_) was used as the nebulizing and sheath gas. Product ion scanning experiments were conducted using ultra-high-purity N_2_ as collision gas. The ESI parameters were set as follows: the capillary voltage is 4.5 kV. The flow rate and temperature of sheath gas were 8 L/min and 350 °C, respectively. The flow rate and temperature of drying gas were 8 L/min and 300 °C, respectively. The pressure of nebulizer gas was 40 psi. The fragmentor voltage was 175 V. The mass analyzer scanned from 100 to 1700 (*m/z*). The Q-TOF acquisition rates were 2 and 3 Hz for full scanning and product ion scanning, respectively. The energies for collision-induced dissociation (CID) experiments were set at 10, 20, 30 and 40 eV, respectively.

The Q-TOF mass spectrometer was tuned in the low mass range (from 100 to 1700 Da) and in the extended dynamic range mode (2 GHz). To ensure the mass accuracy and reproducibility, all MS data were acquired with reference masses at *m/z* 112.9855 and 966.0007 in the negative ESI mode, and at *m/z* 121.0508 and 922.0097 in the positive ESI mode.

## Results

### Comparison of the chemical profiles of sulfur-fumigated and air-dried *Jiegeng* samples

**Fig. 1:**
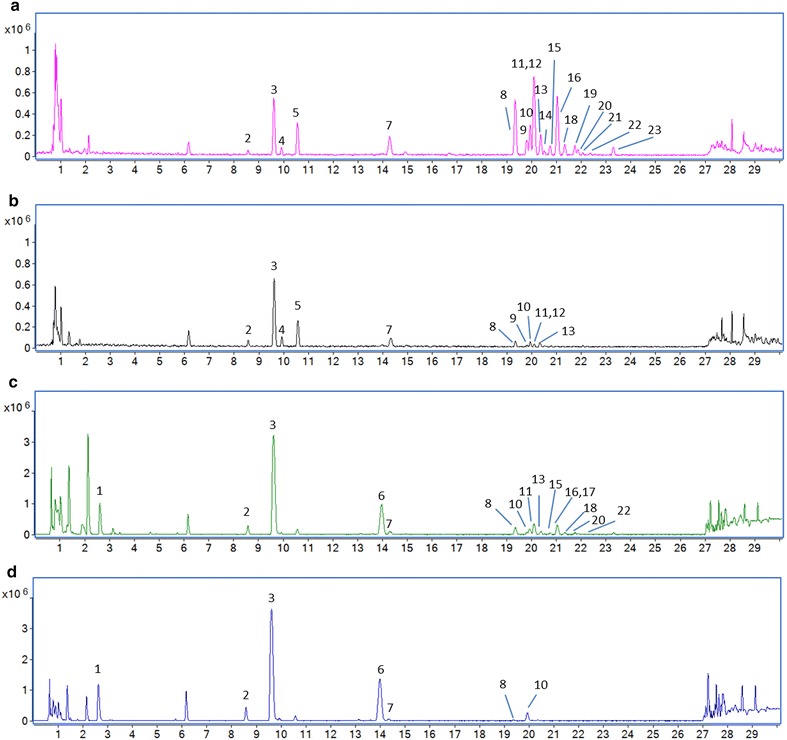
Representative base peak chromatograms of *Jiegeng* samples. **a** Air-dried *Jiegeng* in negative ion mode; **b** Sulfur-fumigated *Jiegeng* in negative ion mode; **c** Air-dried *Jiegeng* in positive ion mode; **d** Sulfur-fumigated *Jiegeng* in positive ion mode

To compare the chemical compositions of sulfur-fumigated and air-dried *Jiegeng* samples, chemical profiling was conducted by UHPLC UHD Q-TOF MS in both positive and negative ion modes. Two batches of air-dried *Jiegeng* samples (FX-JG-01, FX-JG-03) and their corresponding sulfur-fumigated ones (ZX-JG-02, ZX-JG-04) were analyzed using the established UHPLC UHD Q-TOF MS/MS method in this study. Within each sample group (air-dried and sulfur-fumigated), similar results were obtained from the two batches of samples. The results for samples FX-JG-01 and ZX-JG-02, which were from the same batch of fresh *Jiegeng* sample, were described and discussed in detail. The representative base peak ion (BPI) chromatograms of sample FX-JG-01 and ZX-JG-02 were shown in Fig. 1. It was found that the intensity of the major peaks (peaks 5, 7–23) detected in the air-dried *Jiegeng* sample (Fig. 1a, c) were obviously decreased or even disappeared in the corresponding sulfur-fumigated sample (Fig. 1b, d), whereas the peak heights of some components (peaks 1, 2, 3, 4, 6) were slightly higher in the sulfur-fumigated sample than in the air-dried sample. These results suggest that sulfur fumigation caused significant changes in the chemical profile of *Jiegeng*.

### Structural elucidation and identification of chemical components in sulfur-fumigated and air-dried *Jiegeng* samples

**Fig. 2:**
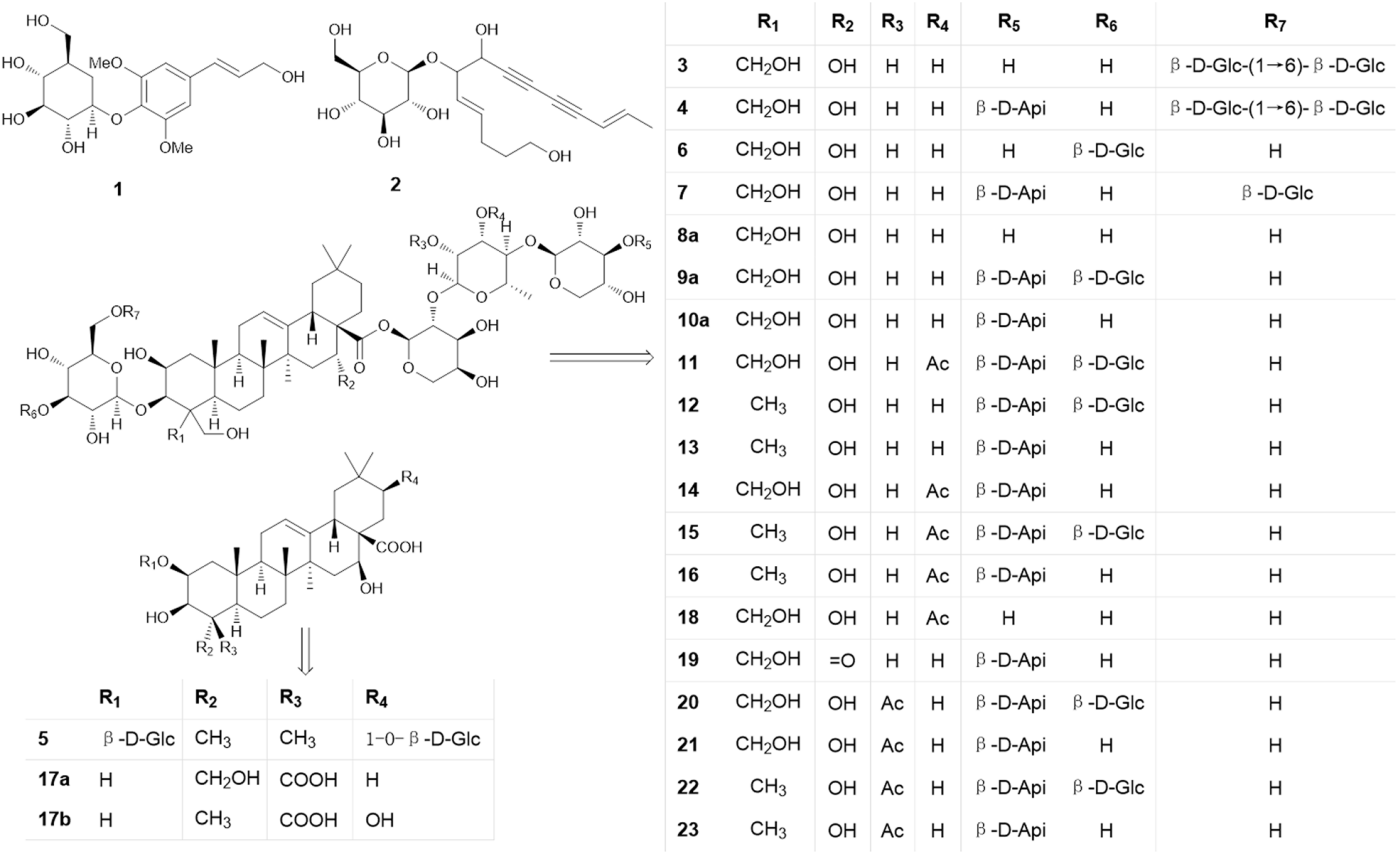
Structures of components identified in sulfur-fumigated and air-dried *Jiegeng* samples

**Fig. 3:**
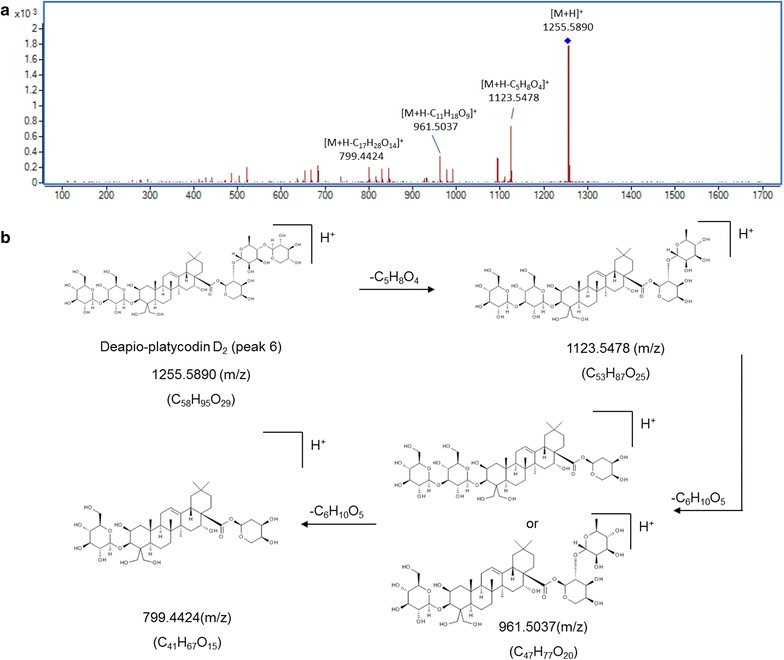
Mass spectra and proposed major fragmentation pathway of deapio–platycodin D_2_ in *Jiegeng* in positive ion mode. **a** Low energy CID mass spectra; **b** Proposed major fragmentation pathway

**Fig. 4:**
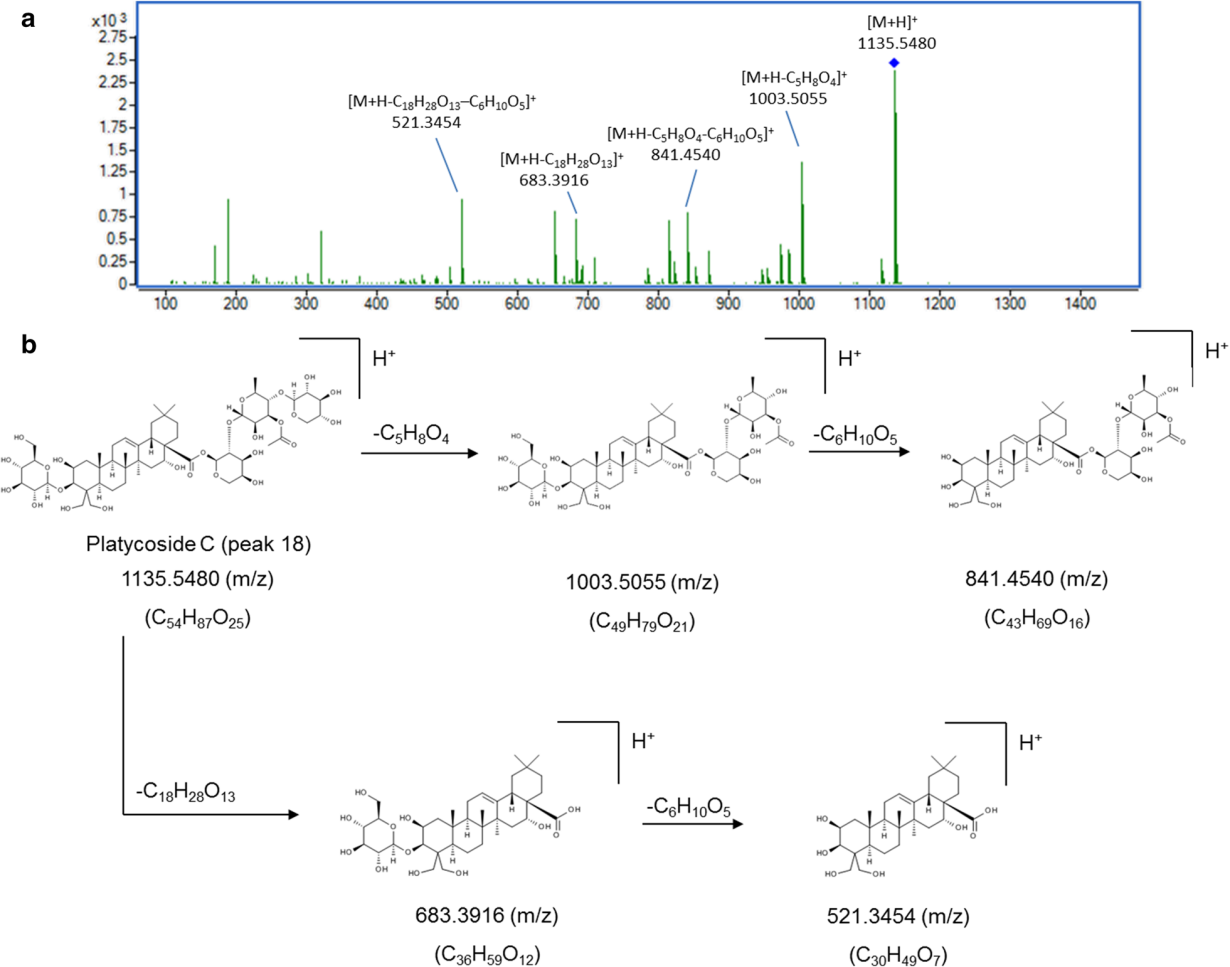
Mass spectra and proposed major fragmentation pathways of platycoside C in *Jiegeng* in positive ion mode. **a** Low energy CID mass spectra; **b** Proposed major fragmentation pathway

**Fig. 5:**
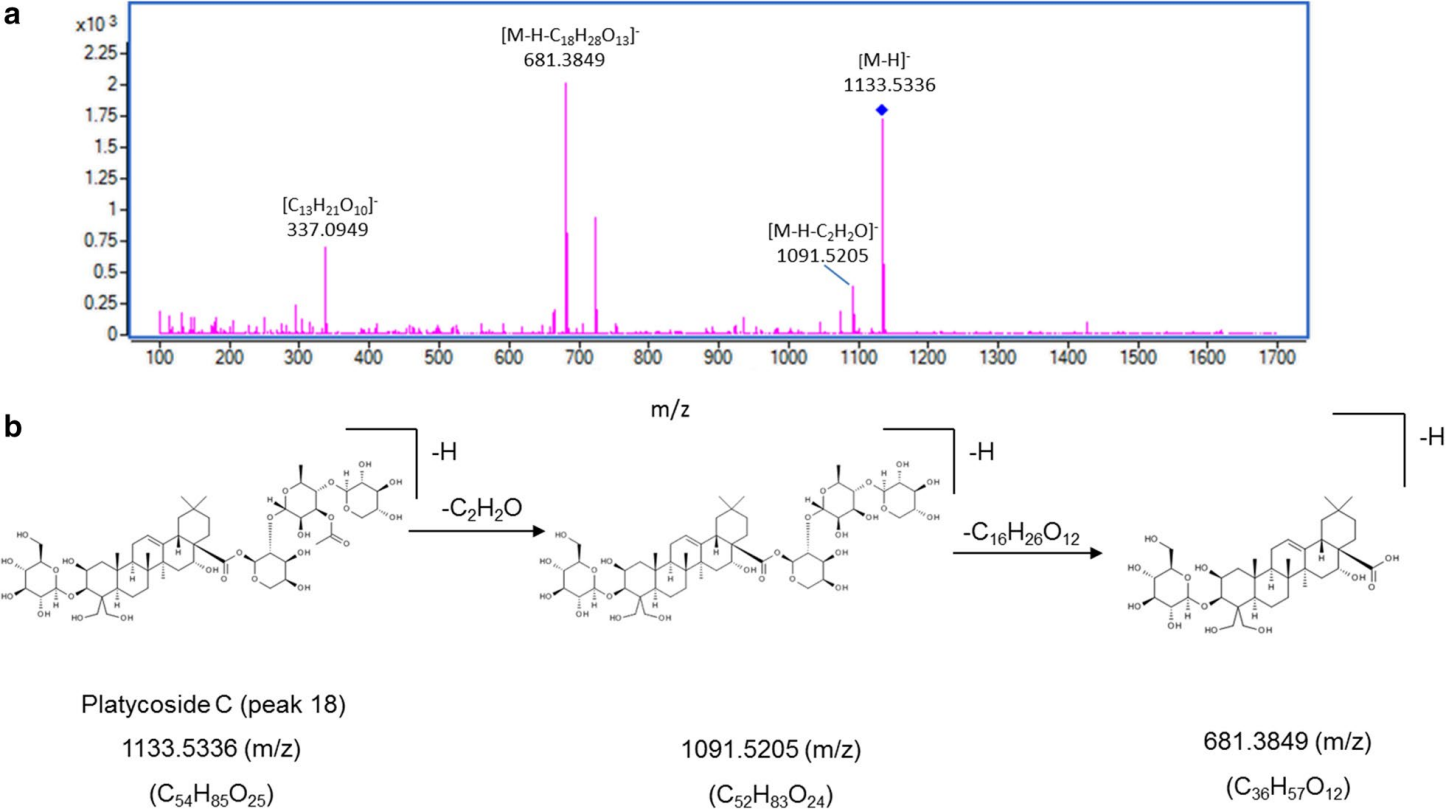
Mass spectra and proposed major fragmentation pathways of platycoside C in *Jiegeng* in negative ion mode. **a** Low energy CID mass spectra; **b** Proposed major fragmentation pathway

**Fig. 6:**
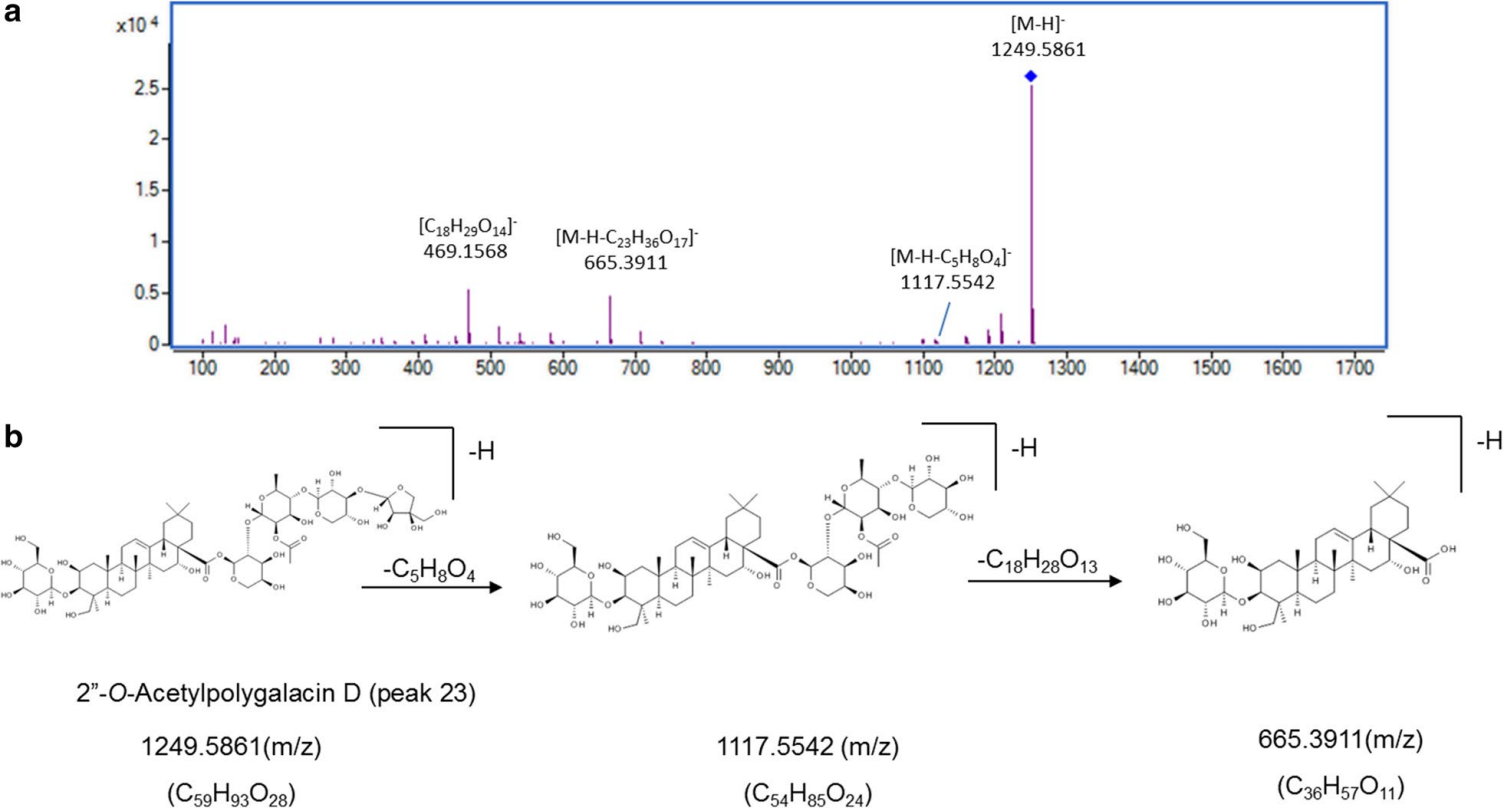
Mass spectra and proposed major fragmentation pathways of 2″-*O*-acetylpolygalacin D in *Jiegeng* in negative ion mode. **a** Low energy CID mass spectra. **b** Proposed major fragmentation pathway

**Fig. 7:**
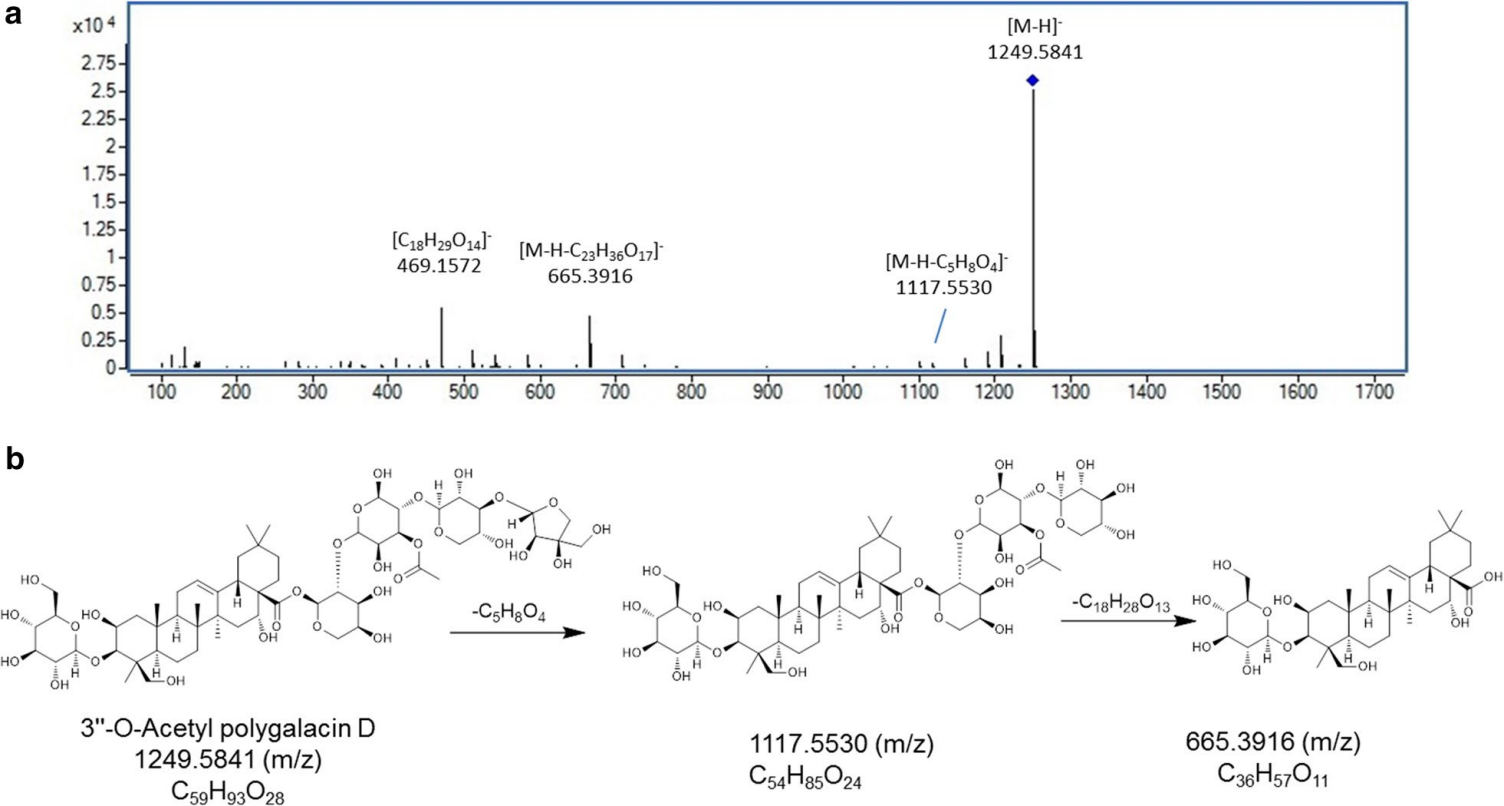
Mass spectra and proposed major fragmentation pathways of 3″-*O*-acetylpolygalacin D in *Jiegeng* in negative ion mode. **a** Low energy CID mass spectra. **b** Proposed major fragmentation pathway

For structural elucidation, the quasi-molecular ion peak of each compound was selected to obtain the product ion spectra with optimal collision energies (Table 1). Peak 2 showed a [M+Na]^+^ ion at *m/z* 419.1664 (C_20_H_28_O_8_) in positive ion mode and the ion could be further fragmented into ion *m/z* 257.1250 [M+Na–C_6_H_10_O_5_]^+^, the fragmented ions corresponded to the loss of one C_6_H_10_O_5_. Moreover, a [M+HCOO]^−^ ion at *m/z* 441.1756 in negative ion mode generated fragments at *m/z* 305.1301 [M–H–C_7_H_6_]^−^ and 215.1107 [M–H–C_6_H_12_O_6_]^−^, resulting from the losses of C_7_H_6_ and one glycosyl moiety, successively. Therefore, peak 2 was tentatively assigned as lobetyolin (**2** in Fig. 2) and its identity was further confirmed by comparing the retention time and mass spectrum of reference standard. For peak 4, precursor ion [M–H]^−^ was observed at *m/z* 1547.6719 (C_69_H_112_O_38_) with product ions at *m/z* 1415.6044, 1283.5692 and 1005.4878 representing the loss of apiosyl, xylosyl and rhamnosyl+arabinosyl moieties attached at C-28 of triterpenoid core, respectively. Therefore, peak 4 was tentatively assigned as platycoside E (**4** in Fig. 2). Peak 6 (Fig. 3) displayed a precursor ion *m/z* 1255.5890 [M+H]^+^ and product ions at *m/z* 1123.5478 [M+H–C_5_H_8_O_4_]^+^, *m/z* 961.5037 [M+H–C_11_H_18_O_9_]^+^ and *m/z* 799.4424 [M+H–C_11_H_18_O_9_–C_6_H_10_O_5_]^+^, and was deduced as deapio-platycodin D_2_ (**6** in Fig. 2). Peak 8 was tentatively assigned as deapio-platycodin D (**8** in Fig. 2) according to the following facts. In positive mode, molecular ion *m/z* 1093.5400 [M+H]^+^ and the fragment ions at *m/z* 961.4949, 799.4453, 683.3975 and 521.3406 were due to glycosidic bond cleavage with sequential loss of xylosyl, rhamnosyl, arabinosyl and glucosyl moieties linked at C-28 and C-3 of the triterpenoid core, respectively. In negative mode, molecular ion *m/z* 1091.5265 [M–H]^−^ and the fragment ions at *m/z* 959.4430, 681.3620 could be sequential loss of xylosyl and rhamnosyl+arabinosyl respectively [11]. Peak 10 showed an [M+Na]^+^ ion at *m/z* 1247.5620 and [M–H]^−^ ion at *m/z* 1223.5669 (C_57_H_92_O_28_). In TOF–MS/MS spectra, fragment ion at *m/z* 705.3831 [M+Na–C_21_H_34_O_16_]^+^ (positive ion mode), and fragment ion at *m/z* 681.3806 [M–H–C_21_H_34_O_16_]^−^ (negative ion mode) were both resulted from the loss of apiosyl+xylosyl+rhamnosyl+arabinosyl moiety. Moreover, by comparing with authentic reference standard, peak 10 was identified as platycodin D (**10** in Fig. 2). For peak 18 (Figs. 4, 5), molecular ion *m/z* 1135.5480 [M+H]^+^ and its product ions *m/z* 1003.5055 [M+H–C_5_H_8_O_4_]^+^, *m/z* 841.4540 [M+H–C_5_H_8_O_4_–C_6_H_10_O_5_]^+^, *m/z* 683.3916 [M+H–C_18_H_28_O_13_]^+^ and *m/z* 521.3454 [M+H–C_18_H_28_O_13_–C_6_H_10_O_5_]^+^ were observed in positive ion mode. Meanwhile, molecular ion *m/z* 1133.5336 [M–H]^−^ and fragment ions *m/z* 1091.5205 [M–H–C_2_H_2_O]^−^ and *m/z* 681.3849 [M–H–C_18_H_28_O_13_]^−^ were observed in negative ion mode. Based on the literature information [14], peak 18 was tentatively assigned as platycoside C (**18** in Fig. 2). For peak 21, precursor ion [M–H]^−^ was observed at *m/z* 1265.5750 (C_59_H_94_O_29_) with product ion [M–H–C_5_H_8_O_4_]^−^ at *m/z* 1133.5379 and [M–H–C_23_H_36_O_17_]^−^ at *m/z* 681.3799 in negative ion mode. Comparing with literature [15], peak 21 was deduced as platycodin A (**21** in Fig. 2). The polygalacin D (peak 14) (**14** in Fig. 2) and 2″-*O*-acetyl polygalacin D (peak 23) (**23** in Fig. 2) are dehydroxylated derivatives of platycodin D and platycodin A, respectively, yielding ion difference of 16 Da between two groups. 2″-*O*-acetyl polygalacin D (peak 23) and 3″-*O*-acetyl polygalacin D (peak 16) (**16** in Fig. 2) are a pair of isomers with molecular weights of 1250 Da and produce the similar product ion spectra (Figs. 6, 7). Using LC-ESI MS/MS analysis and comparing with the chromatographic characteristics in literature [11], the two isomers were tentatively identified.

**Table 1:** Components identified in sulfur-fumigated and air-dried *Jiegeng* samples

No.	Compound name	Formula	RT (min)	[M+H]^+^ mass accuracy (ppm)	[M–H]^−^ mass accuracy (ppm)	Positive ions [M+H]^+^ and proposed CID fragment ions	Negative ions [M–H]^−^ and proposed CID fragment ions	Ref.
1a	Syringin	C_17_H_24_O_9_	2.725	3.33	−	395.1300 [M+Na]^+^ 233.0841 [M+Na−C_6_H_10_O_5_]^+^	−	
2a	Lobetyolin	C_20_H_28_O_8_	8.872	−0.55	1.88	419.1664 [M+Na]^+^ 257.1205 [M+Na−C_6_H_10_O_5_]^+^ 203.0575 [C_6_H_12_O_6_Na]^+^	441.1756 [M+HCOO]^−^ 305.1301 [M−H−C_7_H_6_]^−^ 215.1107 [M−H−C_6_H_12_O_6_]^−^ 179.0565 [C_6_H_11_O_6_]^−^	
3	Platycoside G_1_ (Deapio−platycoside E)	C_64_H_104_O_34_	9.745	5.20	1.20	1417.6425 [M+H]^+^ 503.3338 [C_30_H_47_O_6_]^+^ 485.3247 [C_30_H_45_O_5_]^+^	1415.6327 [M–H]^−^ 1283.5692 [M−H−C_5_H_8_O_4_]^−^ 1005.4907 [M−H−C_16_H_26_O_12_]^−^ 519.3385 [C_30_H_47_O_7_]^−^	[11]
4	Platycoside E	C_69_H_112_O_38_	9.922	−	1.63	−	1547.6719 [M−H]^−^ 1415.6044 [M−H−C_5_H_8_O_4_]^−^ 1283.5692 [M−H−(C_5_H_8_O_4_)_2_]^−^ 1005.4878 [M−H−C_21_H_34_O_16_]^−^	[11]
5	Platycogenin A	C_42_H_68_O_16_	10.661	−	0.60	−	827.4433 [M−H]^−^ 665.3907 [M−H−C_6_H_10_O_5_]^−^	[27]
6	Platycoside A (Deapio−platycodin D_2_)	C_58_H_94_O_29_	13.902	4.70	−	1255.5890 [M+H]^+^ 1123.5478 [M+H−C_5_H_8_O_4_]^+^ 961.5037 [M+H−C_11_H_18_O_9_]^+^ 799.4424 [M+H−C_17_H_28_O_14_]^+^	−	[28]
7	Platycodin D_3_	C_63_H_102_O_33_	14.291	6.00	−0.18	1409.6130 [M+Na]^+^ 867.4480 [M+Na−C_21_H_34_O_16_]^+^	1385.6239 [M−H]^−^ 1253.5975 [M−H−C_5_H_8_O_4_]^−^ 843.4433 [M−H−C_21_H_34_O_16_]^−^	[11, 29]
8a	Deapio−platycodin D	C_52_H_84_O_24_	19.323	3.70	1.31	1093.5400 [M+H]^+^ 961.4949 [M+H−C_5_H_8_O_4_]^+^ 799.4453 [M+H−C_11_H_18_O_9_]^+^ 683.3975 [M+H−C_16_H_26_O_12_]^+^ 521.3406 [M+H−C_16_H_26_O_12_−C_6_H_10_O_5_]^+^	1091.5268 [M−H]^−^ 959.4430 [M−H−C_5_H_8_O_4_]^−^ 681.3620 [M−H−C_16_H_26_O_12_]^−^	[11]
9a	Platycodin D_2_	C_63_H_102_O_33_	19.829	−	−0.74	−	1385.6247 [M–H]^−^ 843.4439 [M–H−C_21_H_34_O_16_]^−^	[30]
10a	Platycodin D	C_57_H_92_O_28_	19.946	4.90	1.16	1247.5620 [M+Na]^+^ 705.3831 [M+Na–C_21_H_34_O_16_]^+^	1223.5669 [M−H]^−^ 681.3806 [M−H−C_21_H_34_O_16_]^−^	[11]
11	3″–*O*-Acetylplatycodin D_2_	C_65_H_104_O_34_	20.088	4.70	2.22	1451.6250 [M+Na]^+^ 867.4011 [M+Na −C_23_H_36_O_17_]^+^	1427.6282 [M−H]^−^ 1295.5884 [M−H−C_5_H_8_O_4_]^−^ 843.4379 [M−H−C_23_H_36_O_17_]^−^ 663.3800 [M−H−C_23_H_36_O_17_–C_6_H_12_O_6_]^−^	[29]
12	Polygalacin D_2_	C_63_H_102_O_32_	20.123	−	0.43	−	1369.6281 [M–H]^−^ 1237.5581 [M–H−C_5_H_8_O_4_]^−^ 827.4438 [M–H−C_21_H_34_O_16_]^−^ 647.3782 [M–H−C_21_H_34_O_16_–C_6_H_12_O_6_]^−^ 541.1819 [C_21_H_33_O_16_]^−^	[29]
13	Polygalacin D	C_57_H_92_O_27_	20.510	−	1.36	−	1207.5738 [M–H]^−^ 665.3726 [M–H−C_21_H_34_O_16_]^−^ 541.1728 [C_21_H_33_O_16_]^−^	[11]
14	Platycodin C (3″-*O*-Acetylplatycodin D)	C_59_H_94_O_29_	20.344	4.30	4.64	1289.5730 [M+Na]^+^ 705.3770 [M+Na–C_23_H_36_O_17_]^+^	1265.5720 [M–H]^−^ 1133.5398 [M–H−C_5_H_8_O_4_]^−^ 681.3842 [M–H−C_23_H_36_O_17_]^−^	[29]
15	3″-*O*-Acetylpolygalacin D_2_	C_65_H_104_O_33_	20.723	4.30	1.25	1435.6290 [M+H]^+^ 851.4342 [M+Na–C_23_H_36_O_17_]^+^	1411.6328 [M–H]^−^ 1279.6025 [M–H−C_5_H_8_O_4_]^−^ 827.4424 [M–H−C_23_H_36_O_17_]^−^ 665.3885 [C_36_H_57_O_11_]^−^ 541.1741 [C_21_H_33_O_16_]^−^ 469.1550 [C_18_H_29_O_14_]^−^	[29]
16	3″-*O*-Acetylpolygalacin D	C_59_H_94_O_28_	21.054	4.60	−0.14	1273.5780 [M+Na]^+^ 689.3757 [M+Na–C_23_H_36_O_17_]^+^	1249.5866 [M–H]^−^ 1117.5502 [M–H−C_5_H_8_O_4_]^−^ 665.3916 [M–H−C_23_H_36_O_17_]^−^ 469.1554 [C_18_H_29_O_14_]^−^	[11]
17a/17b	Platycogenic acid A/platycogenic acid B	C_30_H_46_O_8_	21.166	1.60	−	535.3255 [M+H]^+^ 517.3143 [M+H−H_2_O]^+^ 499.3056 [M+H−(H_2_O)_2_]^+^ 481.2688 [M+H−(H_2_O)_3_]^+^	−	[31]
18	Platycoside C	C_54_H_86_O_25_	21.373	4.40	1.50	1135.5480 [M+H]^+^ 1003.5055 [M+H−C_5_H_8_O_4_]^+^ 841.4540 [M+H−C_5_H_8_O_4_–C_6_H_10_O_5_]^+^ 683.3916 [M+H−C_18_H_28_O_13_]^+^ 521.3454 [M+H−C_18_H_28_O_13_–C_6_H_10_O_5_]^+^	1133.5336 [M–H]^−^ 1091.5205 [M–H−C_2_H_2_O]^−^ 681.3849 [M–H−C_18_H_28_O_13_]^−^ 337.0949 [C_13_H_21_O_10_]^−^	[28]
19	16-Oxoplatycodin D	C_57_H_90_O_28_	21.707	−	1.21	−	1221.5531 [M–H]^−^ 811.1198 [M–H−C_16_H_26_O_12_]^−^ 635.3855 [M–H−C_22_H_34_O_18_]^−^ 541.1765 [C_21_H_33_O_16_]^−^ 409.1354 [C_16_H_25_O_12_]^−^	[14]
20	Platycodin V (2″-*O*-Acetylplatycodin D_2_)	C_65_H_104_O_34_	21.838	5.10	−0.68	1451.6213 [M+Na]^+^ 867.4064 [M+Na −C_23_H_36_O_17_]^+^	1427.6348 [M–H]^−^ 843.4372 [M–H−C_23_H_36_O_17_]^−^ 663.3670 [M–H−C_23_H_36_O_17_–C_6_H_12_O_6_]^−^ 469.1572 [C_18_H_29_O_14_]^−^	[14]
21	Platycodin A (2″-*O*-Acetylplatycodin D)	C_59_H_94_O_29_	22.048	−	3.17	−	1265.5750 [M–H]^−^ 1133.5379 [M–H−C_5_H_8_O_4_]^−^ 681.3799 [M–H−C_23_H_36_O_17_]^−^ 469.1612 [C_18_H_29_O_14_]^−^	[11, 14]
22	2″-*O*-Acetylpolygalacin D_2_	C_65_H_104_O_33_	22.335	5.20	1.29	1435.6290 [M+Na]^+^ 851.4097 [M+Na–C_23_H_36_O_17_]^+^	1411.6374 [M–H]^−^ 827.4408 [M–H−C_23_H_36_O_17_]^−^ 665.3935 [C_36_H_57_O_11_]^−^ 541.1519 [C_21_H_33_O_16_]^−^ 469.1606 [C_18_H_29_O_14_]^−^	[29]
23	2″-*O*-Acetylpolygalacin D	C_59_H_94_O_28_	23.353	−	0.24	−	1249.5861 [M–H]^−^ 1117.5542 [M–H−C_5_H_8_O_4_]^−^ 665.3911 [M–H−C_23_H_36_O_17_]^−^ 469.1568 [C_18_H_29_O_14_]^−^	[11]

### Changes of glycosides of *Jiegeng* after sulfur fumigation

Sulfur fumigation was reported to be able to cause chemical changes in herbs [16, 17]. In this study, chemical changes were observed in *Jiegeng* subjected to sulfur fumigation. Among all the compounds that showed significant changes, 23 constituents were deduced/tentatively identified. Structures of all the compounds were shown in Fig. 2. The peak heights of platycogenin A, platycodin D, platycodin D_2_, platycodin D_3_, polygalacin D, polygalacin D_2_, deapio-platycodin D and 3″-*O*-acetylplatycodin D_2_ in sulfur-fumigated *Jiegeng* were much lower than that in the air-dried sample; peaks of compounds platycodin A, platycodin C, platycodin V, platycoside C, 16-oxoplatycodin D, 2″-*O*-acetylpolygalacin D, 2″-*O*-acetylpolygalacin D_2_, 3″-*O*-acetylpolygalacin D, 3″-*O*-acetylpolygalacin D_2_, and platycogenic acid B disappeared after sulfur fumigation. On the contrary, the contents of syringin, lobetyolin, platycoside E, deapio-platycodin D_2_ and deapio-platycoside E slightly increased after sulfur fumigation.

## Discussion

The decrease and disappearance of the glycoside compounds may be due to the hydrolysis of glycosides catalyzed by sulfurous acid (H_2_SO_3_) which was generated by the combination of SO_2_, the product of sulfur combustion, and the water molecule in fresh *Jiegeng* [3, 15]. For instance, the partial transformation of platycodin D_2_ to deapio–platycodin D_2_ may be caused by H_2_SO_3_-catalyzed hydrolysis of platycodin D_2_. On the other hand, H_2_SO_3_ produced during sulfur fumigation might inactivate glycoside hydrolases and then protect glycosides from hydrolysis in *Jiegeng* [3]. This would partly explain why platycoside E and deapio-platycoside E, in which there are more sugar moieties than in other glycosides of *Jiegeng*, have relatively higher content in sulfur fumigated *Jiegeng* samples than in air-dried *Jiegeng* samples.

It was reported that lobetyolin in Codonopsis Radix (*Dangshen*) could be changed to lobetyolin sulfate, which resulted in significant decrease of lobetyolin content in sulfur-fumigated *Dangshen* [16]. In this study, peak height of lobetyolin in *Jiegen*g increased by sulfur fumigation. However, no peak in the base peak chromatograms of *Jiegeng* samples could be identified as a lobetyolin derivative. It has been shown that lobetyolinin could be hydrolyzed to lobetylin during sulfur fumigation [16]. To determine if this occurred during sulfur fumigation of *Jiegeng*, we extracted the quasi-molecular ion of lobetyolinin in both positive and negative ion modes. A chromatographic peak at 6.83 min in the air-dried sample (Additional file 1: Figure S1) was observed and the fragmentation patterns of this peak agreed with that of lobetyolinin [16]. In positive ion mode, quasi-molecular ion 581.2187 (*m/z)* [M+Na]^+^ (diff = −3.18 ppm) and the corresponding product ions at 419.1494 (*m/z)* [M+Na–C_6_H_10_O_5_]^+^ and 365.0885 (*m/z)* [M+Na–C_14_H_16_O_2_]^+^ could be seen (Additional file 2: Figure S2). In negative ion mode, quasi-molecular ion 603.2279 (*m/z)* [M+HCOO]^−^ (diff = −2.57 ppm) and the corresponding product ions at 467.1782 (*m/z)* [M–H–C_7_H_6_]^−^ were observed (Additional file 3: Figure S3). In sulfur-fumigated sample, the content of lobetyolinin decreased significantly, which can account for the increased content of lobetyolin (Additional file 1: Figure S1). The increased content of lobetyolin might be generated by hydrolysis reaction of lobetyolinin during sulfur fumigation (Additional file 4: Figure S4). Nevertheless, the chromatographic peak of lobetyolinin was masked in the base peak chromatograms of both air-dried and sulfur-fumigated *Jiegeng* samples due to the relative low content of this compound. Figures for the tentative identification of lobetyolinin and the possible mechanism involved in the transformation were provided as Additional files 1, 2, 3 and 4.

Glycosides are the main bioactive constituents of *Jiegeng*, which have been shown to have various bioactivities. Significant decrease and/or loss of these glycosides caused by sulfur fumigation may negatively affect the bioactivities of *Jiegeng*. It has been reported that platycodin D, platycodin D_2_ and platycodin D_3_ have anti-HCV activity [17]; platycodin D is a potent adjuvant of specific cellular and humoral immune responses against recombinant hepatitis B antigen [13], and can reduce pancreatic lipase activity significantly [18]. Whether the decrease in contents of these glycosides during sulfur fumigation will reduce the above mentioned bioactivities of *Jiegeng* remains to be explored.

We also observed that contents of five glycosides (syringe, lobetyolin, deapio-platycodin D_2_, platycoside E and deapio-platycoside E) in *Jiegeng* increased after sulfur fumigation. These glycosides also exhibit various bioactivities. Syringe has immunomodulatory [19] and anti-inflammatory properties [20], can enhance glucose utilization and lower plasma glucose level in rats of insulin deficiency [21], and can alleviate fulminant hepatic failure (FHF) induced by LPS/D-GalN [22]. Lobetyolin has significant antioxidant activity [23] and can activate NF-κB [24]. Deapio-platycodin D_2_ is able to inhibit HCV activity [17]. Platycoside E and deapio-platycoside E have hemolytic activities and adjuvant potentials on the immune responses to Newcastle disease virus-based recombinant avian influenza vaccine (rL-H5) in mice [25], and both showed antioxidant activities [26]. It needs to be investigated if sulfur fumigation would enhance these bioactivities of *Jiegeng*.

## Conclusions

In the present study, an UHPLC UHD Q-TOF MS/MS-based chemical profiling method was developed to reveal sulfur fumigation-caused chemical alterations in *Jiegeng*. We found that sulfur fumigation resulted in significant changes of glycosides, the main bioactive components of *Jiegeng*. Further studies are warranted to determine the mechanism of the transformation of glycosides during sulfur fumigation and whether sulfur fumigation will affect the efficacy and safety of *Jiegeng*.

## Additional files

10.1186/s13020-016-0101-1 Extracted ion chromatograms of lobetiolinin in *Jiegeng* samples. A, air-dried *Jiegeng* in positive ion mode; B, sulfur fumigated *Jiegeng* in positive ion mode; C, air-dried *Jiegeng* in negative ion mode; D, sulfur fumigated *Jiegeng* in negative ion mode.
10.1186/s13020-016-0101-1 Product ion mass spectra and proposed major fragmentation pathways of lobetyolinin in positive ion mode. A, low energy CID mass spectra; B, proposed major fragmentation pathway.
10.1186/s13020-016-0101-1 Product ion mass spectra and proposed major fragmentation pathways of lobetyolinin in negative ion mode. A, low energy CID mass spectra; B, proposed major fragmentation pathway.
10.1186/s13020-016-0101-1 Possible mechanism responsible for the transformation from lobetyolinin to lobetyolin.

## Abbreviations

UHPLC UHD Q-TOF MS/MS: ultra-high resolution quadrupole time-of-flight mass spectrometry
CP: Pharmacopedia of the People’s Republic of China
ESI: electrospray ionization
*m/z*: mass-to-charge ratio
CID: collision-induced dissociation
H_2_SO_3_: sulfurous acid
SO_2_: sulfur dioxide
