# Supplementary figures and images for: Influence of sulfur fumigation on glycoside profile in Platycodonis Radix (Jiegeng)
Source: Chin Med. 2016 Jul 6;11:32. doi: 10.1186/s13020-016-0101-1 (PMC4934009; doi:10.1186/s13020-016-0101-1)

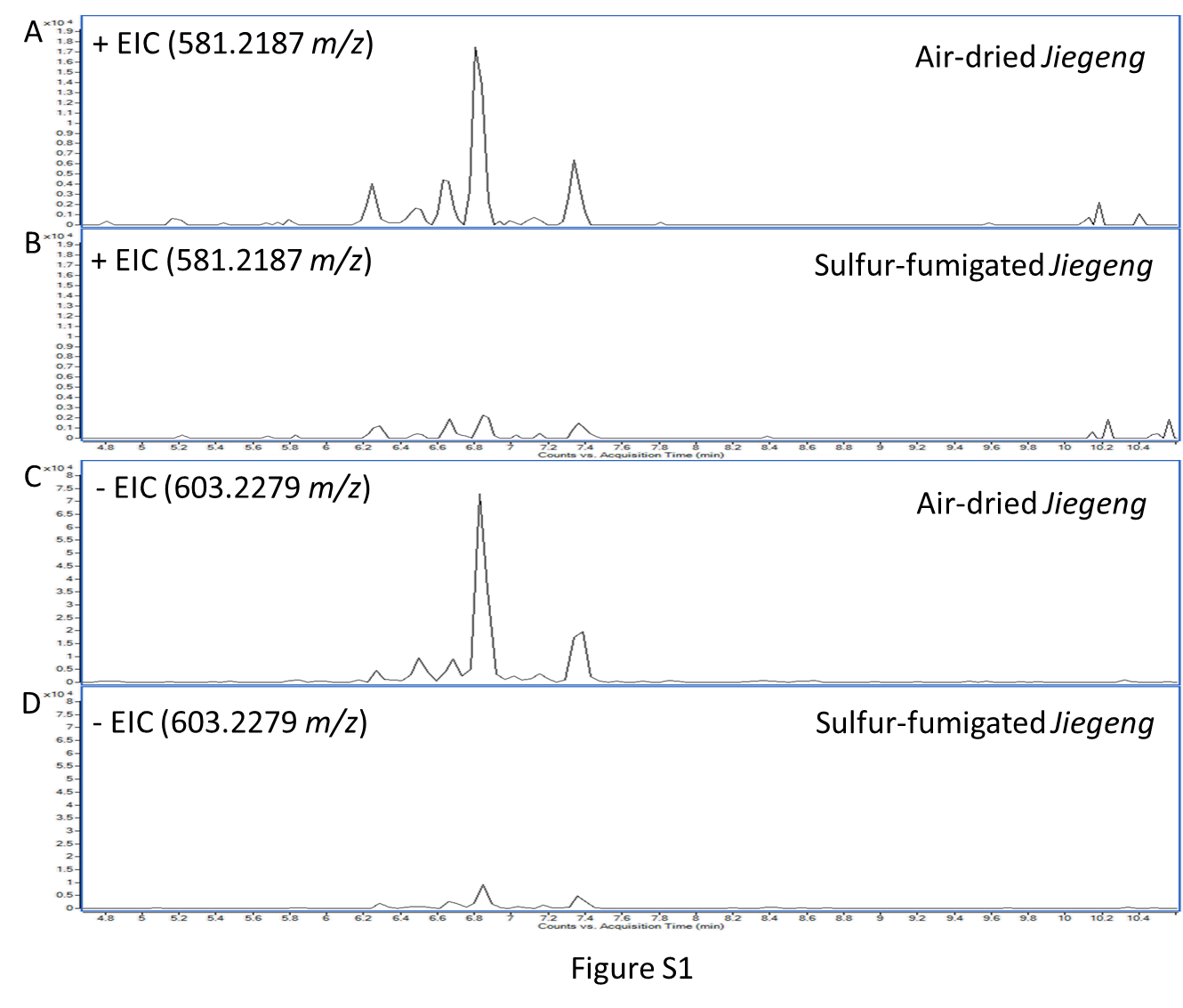

Supplement: Supplementary file 1 — 10.1186/s13020-016-0101-1 Extracted ion chromatograms of lobetiolinin in Jiegeng samples. A, air-dried Jiegeng in positive ion mode; B, sulfur fumigated Jiegeng in positive ion mode; C, air-dried Jiegeng in negative ion mode; D, sulfur fumigated Jiegeng in negative ion mode. [file 13020_2016_101_MOESM1_ESM.png]

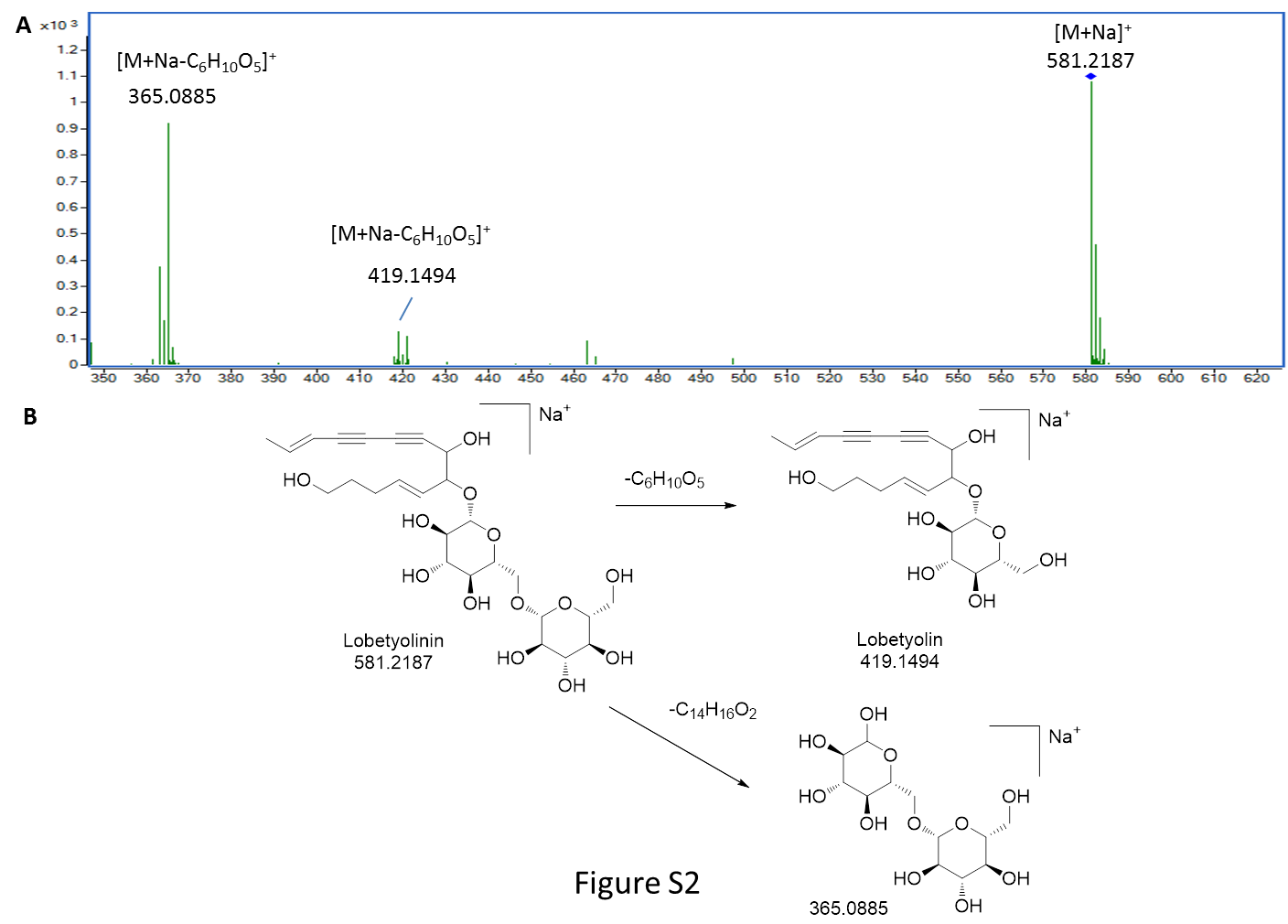

Supplement: Supplementary file 2 — 10.1186/s13020-016-0101-1 Product ion mass spectra and proposed major fragmentation pathways of lobetyolinin in positive ion mode. A, low energy CID mass spectra; B, proposed major fragmentation pathway. [file 13020_2016_101_MOESM2_ESM.png]

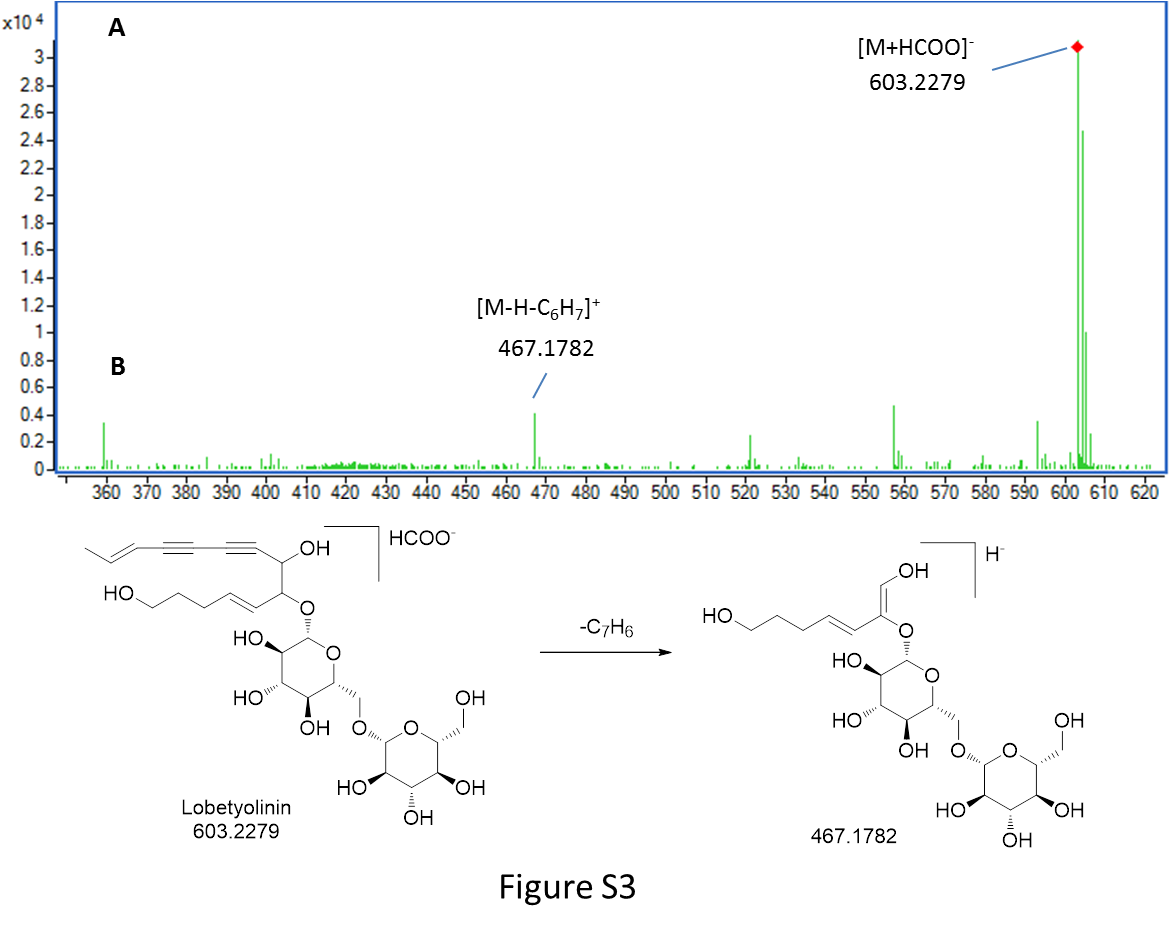

Supplement: Supplementary file 3 — 10.1186/s13020-016-0101-1 Product ion mass spectra and proposed major fragmentation pathways of lobetyolinin in negative ion mode. A, low energy CID mass spectra; B, proposed major fragmentation pathway. [file 13020_2016_101_MOESM3_ESM.png]

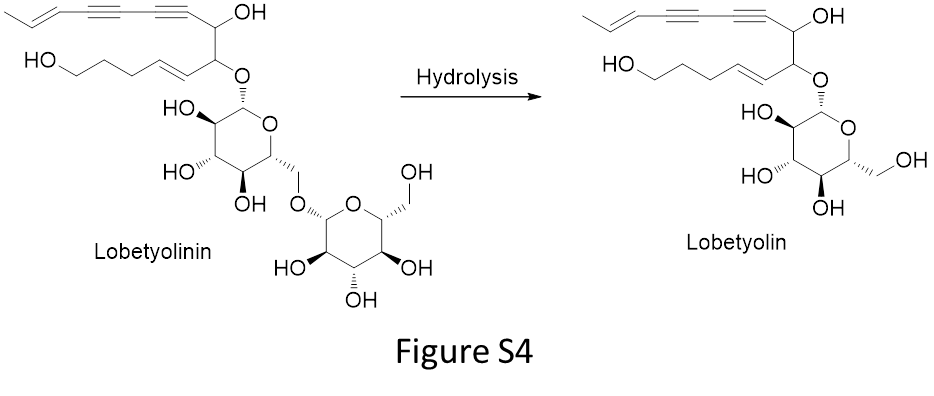

Supplement: Supplementary file 4 — 10.1186/s13020-016-0101-1 Possible mechanism responsible for the transformation from lobetyolinin to lobetyolin. [file 13020_2016_101_MOESM4_ESM.png]
